# Complex III Inhibition-Induced Pulmonary Hypertension Affects the Mitochondrial Proteomic Landscape

**DOI:** 10.3390/ijms21165683

**Published:** 2020-08-08

**Authors:** Joel James, Mathews Valuparampil Varghese, Mikhail Vasilyev, Paul R. Langlais, Stevan P. Tofovic, Olga Rafikova, Ruslan Rafikov

**Affiliations:** 1 Department of Medicine, Division of Endocrinology, University of Arizona College of Medicine, Tucson, AZ 85721, USA; joeljames@deptofmed.arizona.edu (J.J.); mathewsv@email.arizona.edu (M.V.V.); mikhail-vasilyev@uiowa.edu (M.V.); langlais@deptofmed.arizona.edu (P.R.L.); orafikova@email.arizona.edu (O.R.); 2 Vascular Medicine Institute, Department of Pharmacology and Chemical Biology, University of Pittsburgh, Pittsburgh, PA 15213; USA; tofovic@pitt.edu

**Keywords:** pulmonary arterial hypertension, mitochondria, complex III, metabolic dysfunction, fatty acid metabolism

## Abstract

The mitochondria play a vital role in controlling cell metabolism and regulating crucial cellular outcomes. We previously demonstrated that chronic inhibition of the mitochondrial complex III in rats by Antimycin A (AA) induced sustained pulmonary vasoconstriction. On the metabolic level, AA-induced mitochondrial dysfunction resulted in a glycolytic shift that was reported as the primary contributor to pulmonary hypertension pathogenesis. However, the regulatory proteins driving this metabolic shift with complex III inhibition are yet to be explored. Therefore, to delineate the mechanisms, we followed changes in the rat lung mitochondrial proteome throughout AA treatment. Rats treated with AA for up to 24 days showed a disturbed mitochondrial proteome with significant changes in 28 proteins (*p* < 0.05). We observed a time-dependent decrease in the expression of key proteins that regulate fatty acid oxidation, the tricarboxylic acid cycle, the electron transport chain, and amino acid metabolism, indicating a correlation with diminished mitochondrial function. We also found a significant dysregulation in proteins that controls the protein import machinery and the clearance and detoxification of oxidatively damaged peptides via proteolysis and mitophagy. This could potentially lead to the onset of mitochondrial toxicity due to misfolded protein stress. We propose that chronic inhibition of mitochondrial complex III attenuates mitochondrial function by disruption of the global mitochondrial metabolism. This potentially aggravates cellular proliferation by initiating a glycolytic switch and thereby leads to pulmonary hypertension.

## 1. Introduction

Mitochondria are vital organelles of the mammalian cell, directing cell metabolism, cycle, and fate [1]. Disruption in mitochondrial function has been attributed as the root cause of several pathologies, ranging from widespread diseases such as diabetes, obesity, and cardiac complications to rare diseases, including pulmonary arterial hypertension (PAH) [2]. Investigating these diseases has reported metabolic dysfunction to be their common denominator, indicating the importance of mitochondria in controlling cellular metabolism [3]. The metabolic pathways within the mitochondria include the tricarboxylic acid (TCA) cycle, fatty acid metabolism, and amino acid metabolism. These pathways channel reducing equivalents such as nicotinamide adenine dinucleotide (NADH) and flavin adenine dinucleotide (FADH) into the electron transfer chain (ETC) from complex I, complex II, or ubiquinone, and then sequentially from complexes III and IV. These complexes also function to generate a proton gradient across the mitochondrial membrane, which ultimately aids in the generation of ATP at complex V [4,5]. Importantly, the mitochondrial complex III, being at the crossroads to the final step of ATP generation and other metabolic processes, represents a crucial target in cellular energetics in association with metabolic diseases.

We recently demonstrated that chronic inhibition of the mitochondrial complex III with Antimycin A (AA) initiated a cascade of metabolic abnormalities leading to pulmonary hypertension (PH) [6]. Here, pulmonary vasoconstriction and vascular remodeling due to cell proliferation, in association with mitochondrial dysfunction, were determined to be the primary cause of PH [6]. We also demonstrated that vasoconstriction was associated with inhibited electron transfer chain (ETC) and increased nitrosative stress [6]. Indeed, excessive mitochondrial reactive oxygen species (mROS) generation leading to DNA and protein damage has been shown to initiate PH [7]. Interestingly, the complex III is one of the largest ROS generators within the mitochondria and can induce maximal damage as it can release superoxide to both sides of the intermembrane space and the matrix [8,9,10]. Cells with mitochondrial dysfunction are generally directed to apoptosis, but in pathophysiological cases, evading this process leads to uncontrolled proliferation as a result of upregulating glycolysis [11]. This shift, also known as the Warburg effect, has been described in proliferative diseases such as cancer and has been regarded as the primary cause of PAH [3]. In addition to this, our recent work showed that recurrent inhibition of the mitochondrial complex III induced a glycolytic switch in cells with pulmonary vascular cell proliferation [6]. However, the mechanism of how complex III inhibition leads to a metabolic shift in PH is not yet elucidated. Therefore, to bridge this gap, we attempted to address the following questions. Firstly, how does complex III inhibition alone initiate mitochondrial dysfunction, and secondly, what metabolic factors trigger a switch following mitochondrial dysfunction? To evaluate these questions, we performed quantitative proteomics at various time points of complex III inhibition in isolated lung mitochondria. Thus, understanding the specific proteomic changes in the mitochondria in the course of metabolic switch preceding PAH with complex III inhibition presents an opportunity to develop appropriate therapeutic targets.

## 2. Results

### 2.1. Complex III Inhibition Significantly Disrupted the Mitochondrial Proteome

**Figure 1:**
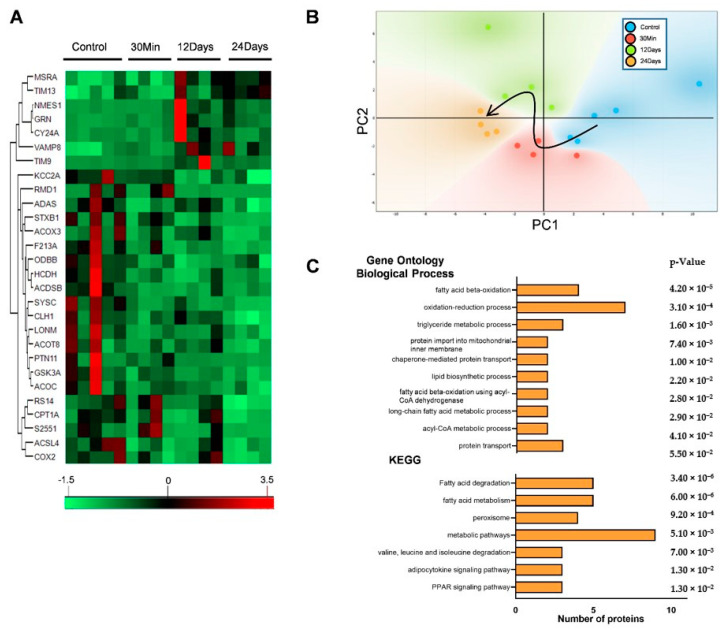
Mitochondrial proteome was significantly altered with complex III inhibition. The quantitative proteomics data were processed using the Perseus software package. (**A**) Unbiased hierarchical clustering of the 28 significantly affected mitochondrial proteins with complex III inhibition. (**B**) Unbiased principal component analysis (PCA) of the 28 significantly affected proteins revealed that the protein expression differences of the individual biological samples within each group were consistent, and no outliers were detected. The black arrow in the PCA plot represents the trajectory of changes by the treatment time (*n* = 4–5). Functional annotation using Database for Annotation, Visualization, and Integrated Discovery (DAVID) with significant pathways arranged according to the *p*-value. (**C**) Representation of significantly enriched processes in the GO-BP (Gene Ontology biological process) and pathways in Kyoto Encyclopedia of Genes and Genomes (KEGG).

Previous studies by our group suggest that initial mitochondrial dysfunction as a result of complex III inhibition was involved in the early changes associated with PAH. We showed that increased right ventricle systolic pressure (RVSP), pulmonary vasoconstriction, and metabolic reprogramming was associated with a glycolytic shift and decreased mitochondrial metabolism [6]. In the present study, we therefore investigated the molecular events following the changes in the mitochondrial proteome to understand the mechanism of this metabolic shift. To achieve this, we followed quantitative mitochondrial proteomics in the lungs of rats treated with AA for 30 min, 12 days, and 24 days in comparison with an untreated group. We selected these time points on the basis of our previous observation that the PAH was more severe at days 12 and 24, representing chronic disease. The time point 30 min was selected as an initial treatment reference point. Quantitative proteomic analysis of the protein expression changes between the control and AA treatment groups identified a total of 1937 proteins. Analysis of these proteins with the Progenesis software package showed that the expression level of 171 proteins was significantly different between the 4 groups (0, 30 min, 12 days, 24 days). Following this, analysis of the significant proteins using gene ontology-based assessment (GO) for functional category co-occurrence using the functional annotation tool DAVID showed that 28 proteins were associated with the GO cellular component term “mitochondrion” (Figure 1A). Unbiased principal component analysis (PCA) of these mitochondrial proteins showed that the expression profile of the four groups showed significant differences and clustering by groups, according to their treatment time (Figure 1B). We then performed a second analysis of the significantly affected mitochondrial proteins for biological process enrichment using DAVID [12]. The result of the GO analysis was sorted according to the *p*-values, and all the significant processes associated with the GO biological process (GO-BP) and KEGG pathways resulting from the analysis in DAVID are represented in Figure 1C. The GO-BP revealed that a majority of the significant processes (7/10 processes) affected were related to fatty acid/lipid metabolism. Following this, we observed that pathways related to protein import and metabolism showed a significant association with the GO-BP. Strikingly, we also found that the highest number of proteins associated with the GO-BP was with the process of oxidation reduction, indicating a potential redox imbalance. The association with KEGG pathways also showed that fatty acid metabolism was significantly affected. Finally, we also observed that the occurrence of the highest number of proteins was associated with the process for “metabolic pathways” in the KEGG pathway analysis (Figure 1C). This indicated that the inhibition of complex III significantly deranged mitochondrial metabolism.

### 2.2. Complex III Inhibition Significantly Dysregulated Mitochondrial Fatty Acid and TCA Metabolism

**Figure 2:**
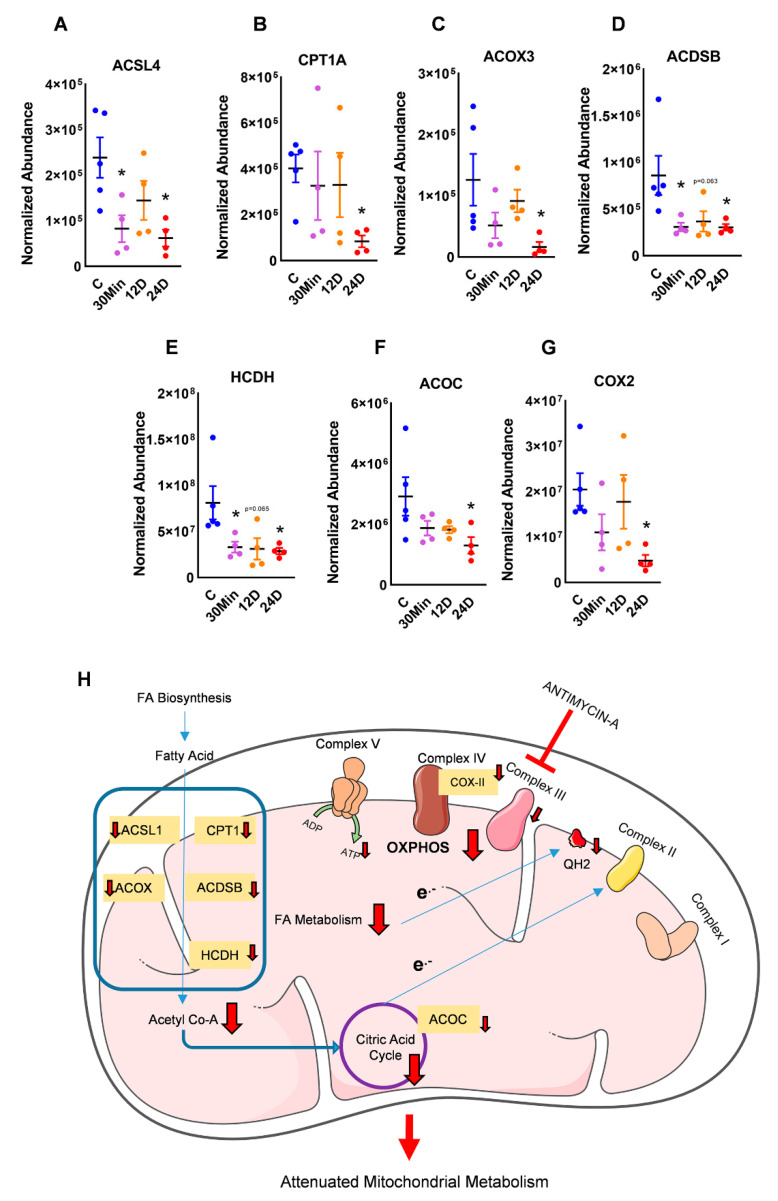
Complex III inhibition disrupted mitochondrial metabolism. Proteins in the fatty acid oxidation pathway: (**A**) carnitine palmitoyltransferase 1A (CPT1A), (**B**) acyl-CoA synthetase long chain family member (ACSL), (**C**) acyl-CoA oxidase 3 (ACOX3), (**D**) short/branched chain-specific acyl-CoA dehydrogenase (ACDSB), and (**E**) hydroxyacyl-coenzyme A dehydrogenase (HCDH) were found to be significantly decreased with complex III inhibition. The tricarboxylic acid (TCA) cycle was potentially disrupted, as observed by decreased (**F**) aconitase (ACOC). Mitochondrial electron transfer chain (ETC) complex IV subunit (**G**) cytochrome c oxidase-II (COX2) was found to decrease with complex III inhibition. (**H**) Decreased fatty acid oxidation would decrease the supply of acetyl-CoA into the TCA cycle. This, in tandem with decreased aconitase, would attenuate the transfer of reducing equivalents to the mitochondrial ETC. Additionally, decreased complex IV activity would consequently stunt ATP generation, leading to mitochondrial dysfunction and a potential glycolytic switch. Red ‘T’ arrow shows inhibition, red arrows show downregulation and all other arrows show transfer (mean ± SEM, *n* = 4-5, * vs. 0h treatment, *p* < 0.05, *t*-test).

Our proteomic data, in conjunction with DAVID, showed that crucial metabolic pathways were affected with complex III inhibition. Upon investigation, the pathways that showed a severe impairment were the fatty acid pathways, TCA cycle, and ETC. Carnitine palmitoyltransferase 1A (CPT1A) is an essential long-chain fatty acid shuttle. CPT, in conjunction with Acyl-CoA synthetase long-chain family member (ACSL), is shown to transfer fatty acids across the mitochondrial membrane [13]. Following this, FA breakdown is catalyzed by acyl-CoA oxidase 3 (ACOX3), hydroxyacyl-coenzyme A dehydrogenase (HCDH), and short/branched chain-specific acyl-CoA dehydrogenase (ACDSB). In our study, we found CPT1A, ACSL4, ACOX3, ACDSB, and HCDH, which are all major FA breakdown proteins that are significantly downregulated in a time-dependent manner with complex III inhibition (Figure 2A–E).

The end product of fatty acid oxidation (FAO) is acetyl-CoA, which feeds into the TCA cycle. Interestingly, we found aconitase (ACOC), a key enzyme that catalyzes the conversion of citrate to iso-citrate in the TCA cycle, to be downregulated with complex III inhibition (Figure 2F), indicating that the citric acid cycle could be impaired in tandem with the FA breakdown. The reducing equivalents from both β-oxidation and the TCA cycle eventually entered the electron transport chain of the mitochondria. With dysregulated FA breakdown and TCA, we also observed an impairment of cytochrome c oxidase-II (COX2), a complex IV subunit (Figure 2G). This, occurring in conjunction with the inhibition of complex III, could essentially stunt ATP production, forcing the cell to channel its metabolic needs from other sources such as increasing glycolysis (Figure 2H).

### 2.3. Complex III Inhibition Impaired Amino Acid Metabolism and Protein Repair

**Figure 3:**
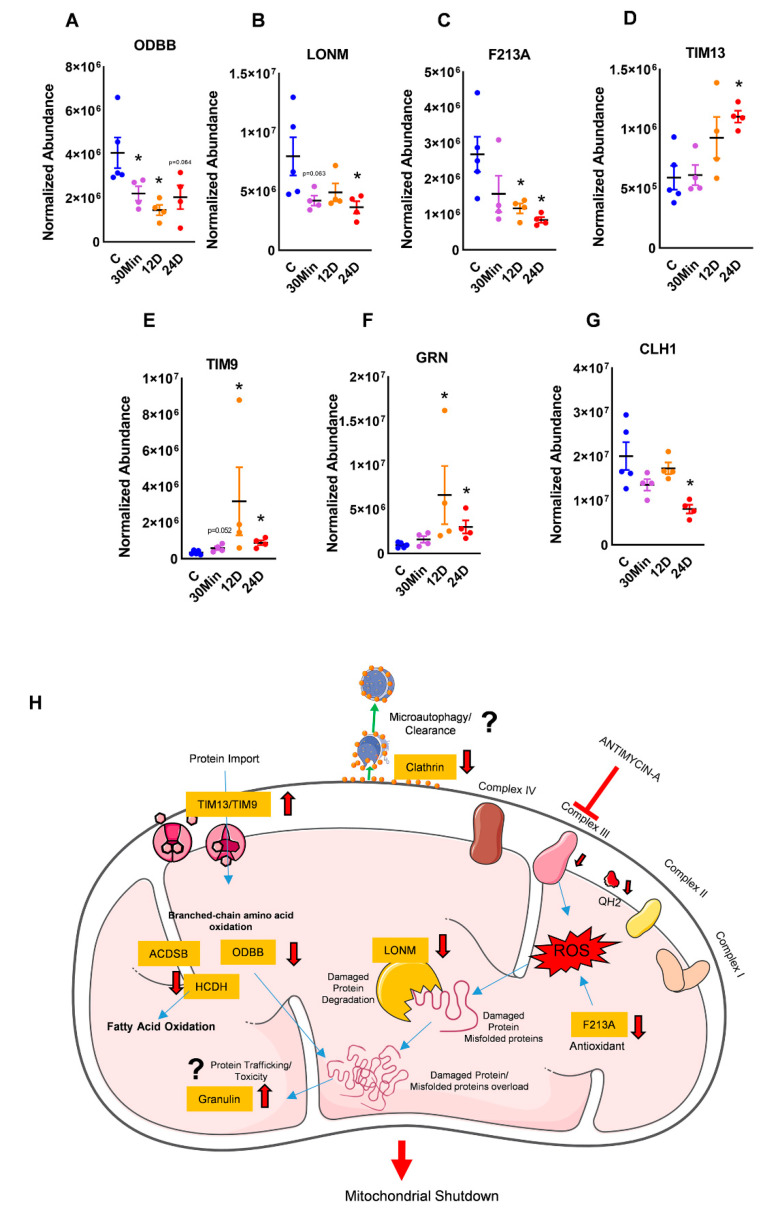
Complex III inhibition instigated dysfunctional protein metabolism and folding with oxidative stress. (**A**) 2-Oxoisovalerate dehydrogenase subunit beta (ODBB), a protein that metabolizes BCCAs or branched-chain amino acids, was found to be significantly downregulated in complex III-inhibited groups. (**B**) Lon protease homolog (LONM), a protein that repairs damaged polypeptides and (**C**) peroxiredoxin-like 2A (PRXL2A or F213A), an antioxidant protein, were found to be decreased in complex III-inhibited groups. (**D**) TIM13 and (**E**) TIM9 peptide transport proteins were significantly upregulated with complex III inhibition. (**F**) Granulin (GRN), a trafficking protein, and (**G**) clathrin (CLH1), a protein assisting microautophagy, were significantly decreased with complex III inhibition. (**H**) Decreased amino acid metabolism in conjunction with decreased protein repair and oxidative damage clearance would cause a toxic mitochondrial overload. This, in conjunction with increased inflammation and reduced microautophagy, could potentially shut down mitochondrial function. Red ‘T’ arrow shows inhibition, red arrows show downregulation and all other arrows show transfer (mean ± SEM, *n* = 4-5, * vs. 0h treatment, *p* < 0.05, *t*-test).

The mitochondria are known to metabolize amino acids and feed their breakdown products into the TCA cycle [14]. BCCAs or branched-chain amino acids are broken down by ODBB (2-oxoisovalerate dehydrogenase subunit beta) [15]. We found ODBB to be significantly downregulated with the inhibition of complex III (Figure 3A), indicating that there could have been a potential build up of amino acids within the mitochondria. Secondly, we found a decrease in Lon protease homolog (LONM), a protein that maintains homeostasis by degrading damaged or misfolded polypeptides and prevents mitochondrial protein aggregation (Figure 3B) [16]. In addition to this, the decrease in peroxiredoxin-like 2A (PRXL2A or F213A), a protein with antioxidant and anti-oxidative stress functions in the mitochondria, could supplement the additional buildup of damaged peptides (Figure 3C) [17]. Finally, we also found an increase in mitochondrial import translocase inner membrane subunit (TIM13), and TIM9, proteins that facilitate peptide transport into the mitochondria, leading to toxic protein accumulation in the mitochondria (Figure 3D,E). Granulin (GRN) is a protein that facilitates protein trafficking and is also implicated in inflammation [18]. Clathrin (CLH1) is an organelle surface protein that aids in microautophagy and is required for organelle maintenance [19,20]. With complex III inhibition, we observed an increase in granulin and a decrease in clathrin (Figure 3F,G). This could imply that this misbalance could contribute to overall toxicity in the mitochondria, leading to mitochondrial shut down, forcing a switch of energy to other sources (Figure 3H).

## 3. Discussion

**Figure 4:**
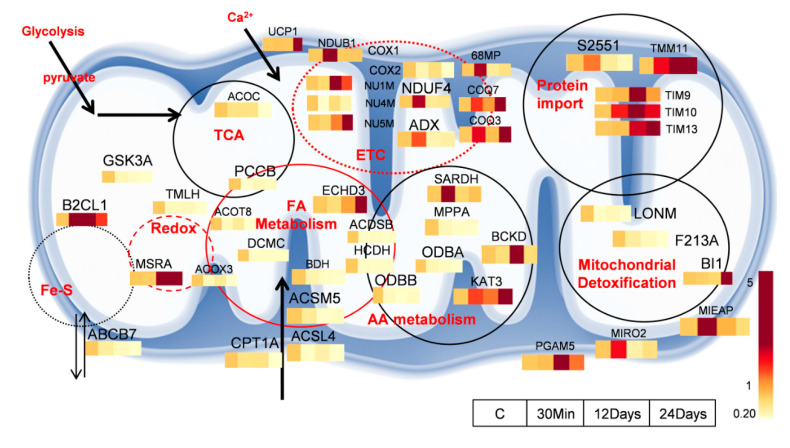
Overview of proteins involved in dysfunctional pathways in chronic mitochondrial complex III inhibition. Dysfunctional fatty acid metabolism, TCA cycle, and ETC can significantly decrease mitochondrial energy production. Pathways of amino acid metabolism converge closely with fatty acid metabolism to further decrease mitochondrial metabolism. Increased protein import and decreased toxic clearance of damaged proteins can ultimately lead to mitochondrial dysfunction, triggering a glycolytic switch.

The mitochondria are a hub of major metabolic processes and also regulate cell fate [21]. However, several aspects of metabolic control by the mitochondria in the pathogenesis of diseases are still not well defined. We previously demonstrated that the inhibition of complex III initiates a cascade of abnormalities in mitochondrial respiration that finally results in a metabolic switch to glycolysis, ultimately leading to PAH [6]. In the present study, with complex III inhibition, we observe a dynamic disruption in several mitochondrial processes. Figure 4 represents proteins with major fold changes in vital mitochondrial processes.

We observed a disruption in primary mitochondrial processes such as the TCA cycle and fatty acid metabolism. Following this, we found a significant disruption in the complexes of the electron transport chain. This impaired energy production was further disrupted by decreased protein expression in several proteins within the amino acid metabolic pathway. These irregularities led to increased mitochondrial toxicity and were culminated in dysfunctional mitochondrial detoxification processes, leading to impaired or stunted mitochondrial function (Figure 4), triggering a glycolytic switch.

In addition to glycolysis, mitochondrial fatty acid β-oxidation (FAO) is an aerobic process that also generates acetyl-CoA and, ultimately, ATP [22]. To facilitate FAO, fatty acids are transported across the mitochondrial membrane by CPT in conjunction with ACSL. This prepares the FAs to be oxidized by the mitochondria through the sequential steps in β-oxidation [23]. Therefore, the efficiency of FAO relies on the efficient transfer of electrons from β-oxidation to NADH and QH2, and ultimately to generate ATP in the ETC. Disruption at any step within this chain of events would potentially create a bottleneck in energy production. Importantly, in this study, with complex III inhibition, we found a significant change in the expression profile of proteins associated with the fatty acid metabolism pathways. This decrease in FAO proteins could only be explained by a direct association of the complex III to the fatty acid complexes [24]. Components of the trifunctional protein (TFP) tetramer (a complex of fatty acid oxidation proteins: enoyl-CoA, hydratase, 3-hydroxyacyl-CoA dehydrogenase, and 3-ketoacyl-CoA thiolase) were shown to directly interact with the mitochondrial oxidative phosphorylation proteins to transfer reducing equivalents into the ETC [23]. Hydroxyacyl-CoA dehydrogenase (HCDH), which generates NADH in the process of converting 3- hydroxyacyl-CoA to 3-ketoacyl-CoA, has been shown to interact both with complex I and III and was found to be significantly decreased in our study [23]. This decrease in HCDH in correlation with complex III, a crucial interacting partner with the FAO complexes, could explain the decrease in FAO. In addition to the FAO and OXPHOS interactions, the discovery of mitochondrial supercomplexes (association of ETC components with each other) would explain why complex III inhibition also regulates the expression of COX2, a complex IV protein that was found to be decreased in our study [25]. Although increased FA metabolism has been described in proliferative cells, conversely, decreased FA metabolism has also been reported in PAH [26,27]. Interestingly, in certain diseases with mitochondrial dysfunction, it was demonstrated that blunted fatty acid oxidation was compensated by enhanced glycolysis [28]. Therefore, we surmise that with complex III inhibition, a deficient FAO could induce a glycolytic switch in the cells and therefore lead to PAH.

Mitochondrial dysfunction is known to generate pathological concentrations of reactive oxygen species, which are known to damage lipids, proteins, and DNA. Importantly, mROS is shown to regulate the flux of mitochondrial metabolism [29]. Therefore, redox regulation and homeostasis within the mitochondria play a vital role in controlling the initiation of pathophysiological processes. In our study, with complex III inhibition, we found decreased expression of a redox regulatory protein, F213A, which also regulates inflammation via the mitogen-activated protein kinase (MAPK) pathway [30]. This suggests that the mitochondrial environment under complex III inhibition is highly oxidative. An oxidative environment could deactivate several vital mitochondrial redox enzymes and also cause protein damage and misfolding with endoplasmic reticulum stress [31]. Indeed, has been demonstrated that there is a crosstalk between mROS and the endoplasmic reticulum [32]. Increased mROS with endoplasmic reticulum (ER) stress could, therefore, potentially lead to excessive cellular proliferation and exacerbate PAH [33]. We also found that complex III inhibition elicits an impairment of the mitochondria by affecting the expression of LONM, a protein that degrades damaged and misfolded proteins and therefore maintains mitochondrial homeostasis [16]. Recent research has shown that LONM is emerging as a master regulator of mitochondrial functions [34]. In addition to the proteolytic activity of LONM, it is demonstrated that LONM also has the ability to function as a molecular chaperone and protects mitochondrial proteins from oxidative damage and aggregation [35,36]. Interestingly, LONM is shown to chaperone the assembly of the mitochondrial ETC, making it an important target to study in mitochondrial dysfunction [37,38]. Although, with regards to PAH, LONM has not been well explored, it is demonstrated that decreased LONM activity is associated with mitochondrial dysfunction and metabolic disease [39]. Moreover, decrease in LONM could contribute to unfolded protein stress [40]. Altogether, the decrease in LONM that we observed with complex III inhibition could potentially increase misfolded proteins, lead to protein aggregation, induce mitochondrial dysfunction, and contribute to the glycolytic switch in PAH [6].

Misfolded proteins and protein aggregation could lead to mitochondrial toxicity, and therefore clearance of damaged mitochondria by the mitophagy process is essential to maintain cellular function [41]. In our study, we found that impaired amino acid breakdown as a result of decreased ODBB and increased protein transport by TIM9 and -13 would only increase the accumulation of damaged proteins and mitochondrial toxicity. Moreover, the increase in TIM9 and -13 have been associated with proliferative diseases such as cancer [42]. Importantly, an increase in damaged proteins with insufficient mitochondrial repair through mitochondrial fission/fusion or mitophagy has been shown to initiate PAH [6,43]. As observed in our study, with complex III inhibition, the decrease in clathrin and increase in granulin could cause a potential reduction in the clearance process of damaged proteins and an increase in inflammation. Granulin is derived from its precursor protein, progranulin, and has been shown to have reciprocal functions [44]. While progranulin promotes mitophagy, its decrease was shown to impair autophagy, and although progranulin is anti-inflammatory, granulin is pro-inflammatory [44,45,46]. Therefore, in complex III inhibition, with increased granulin in the cell, we speculate that the mitophagy process could be decreased. Indeed, granulin has been shown to be elevated in PAH patients [47]. Overall, with complex III inhibition, a decline in processes that “clean up” or repair the mitochondria could ultimately lead to dysfunctional mitochondria and force the cell to switch to glycolytic metabolism and accordingly meet its energy demands.

## 4. Materials and Methods

### 4.1. Animals

The rats were bred in-house at the University Animal Care at the University of Pittsburgh. The animals were kept in a 12 h light–dark cycle and received standard rodent food and water ad libitum. Female rats (200–250g) obtained from Charles River (Wilmington, MA, USA) were used for this study. All experimental procedures were performed according to the University of Pittsburgh institutional guidelines for animal welfare, and the Animal Care and Use Committee approved experimental protocols. AA treatments were carried out as previously described for 30 min, 12 days, and 24 days [6]. For the Antimycin A (AA) injections, the animals were anesthetized (pentobarbital, 45 mg/kg i.p.) and randomized to either receive AA or vehicle. The PE-50 (polyethylene) catheter was inserted in the right jugular vein and advanced into the right atrium for i.v. AA injection. For 30 min AA treatment, we injected 30 μL of 0.35 mg/kg AA dissolved in 55% ethanol, 45% of 0.9% saline as a slow bolus. This dose was determined to be the lowest effective dose tolerated by the rats. The bolus injection was performed directly into the right atrium to maximize the exposure of the pulmonary vasculature to AA and minimize the effects on the left ventricle and other organs. For the chronic studies, the PE-50 catheter was placed into the right jugular vein, fixed to the sternocleidomastoid muscle, and advanced subcutaneously to the back of the neck through the incision between the scapulae. The ventral neck incision was closed with the wound clips and the dorsal, with 4–0 silk suture to secure the exterior part of the catheter in place, was used for chronic AA injections on days 3 and 6.

### 4.2. Mitochondrial Isolation

Mitochondrial isolations were carried out as previously described [48]. In brief, rat lungs were excised and homogenized in the Fisher homogenizer 850 (10,000 RPM, 1min) in ice-cold isolation buffer (50 mM Tris/HCl (pH 7.4), 250 mM sucrose, 5 mM ethylenediaminetetraacetic acid (EDTA) and protease inhibitors) followed by gentle shaking in the UltraCruz Shaker (0 °C, 10 min). The samples were centrifuged at 700× *g*, 7800× *g*, and 12,000× *g* sequentially to pellet the mitochondria. The mitochondria were snap-frozen and stored at −80 °C until further analyses.

### 4.3. Mass Spectrometry

#### 4.3.1. In-Solution Tryptic Digestion of Isolated Mitochondria

To determine alterations in the mitochondrial proteome accompanying complex III inhibition, we added 50 µL of membrane solubilization buffer (cat# 1862783, Thermo Scientific, Rockford, IL, USA) to isolated mitochondria and incubated the mixture on ice for 30 min. A total of 40 µL of supernatant (clarified) was collected and subjected to in-solution tryptic digestion. Sample volumes were adjusted to 100 µL with membrane solubilization buffer containing dithiothreitol (DTT) (5mM final concentration). Following this, the samples were incubated at 56 °C for 30 min and later brought to room temperature (10min). Again, they were incubated with 15mM acrylamide for 30 min (room temperature, protected from light). Six volumes of pre-chilled 100% acetone were added to the samples and they were then incubated (1 h at −20 °C). This precipitated the proteins. This was followed by centrifugation (16,000× *g*, 10 min, 4 °C). Samples were then vortexed after the addition of 400 µL of pre-chilled 90% acetone. The samples were then centrifuged at 16,000× *g* for 5 min at 4 °C. The residual acetone was discarded, and the protein pellets were air-dried (2–3 min). The protein pellet was resuspended in 50 µL of digestion buffer (Pierce MS Sample Prep Kit for Cultured Cells, cat# 84840, Thermo Scientific), and 2 µg of Lys-C was added to each sample and incubated (37 °C, 2 h, shaking at 300rpm). Subsequently, 50 µL of 50 mM ammonium bicarbonate and 2 µg of trypsin was added to each sample and incubated (37 °C, overnight, shaking at 300 rpm). To halt the digestion, we froze the samples at −80 °C for 10 min. The samples were dried and resuspended in 15 µL 0.1% heptafluorobutyric acid (HFBA)/4% formic acid (FA) (*v/v*) and incubated at room temperature for 15 min. The samples were desalted, as previously described [49]. The dried peptides were resuspended in 6 µL of 0.1% FA (*v/v*) and sonicated for 1 min, and then 2.5 µL of the final sample was analyzed by mass spectrometry.

#### 4.3.2. Mass Spectrometry and Data Processing 

HPLC–ESI–MS/MS was carried out in positive ion mode on a Thermo Scientific Orbitrap Fusion Lumos tribrid mass spectrometer fitted with an EASY-Spray Source (Thermo Scientific, San Jose, CA). NanoLC was performed using a Thermo Scientific UltiMate 3000 RSLCnano System with an EASY Spray C18 LC column (cat. # ES803, Thermo Scientific Rockford, IL, USA, 50 cm × 75 μm inner diameter, packed with PepMap RSLC C18 material, 2 µm), a loading phase for 15 min at 0.300µL/min, and a mobile phase with a linear gradient of 1–34% Buffer B for 119 min at 0.220 µL/min, followed by a step to 95% Buffer B over 4 min at 0.220 µL/min, hold 5 min at 0.250 µL/min, and then a step to 1% Buffer B over 5 min at 0.250 µL/min and a final hold for 10 min (total run 159 min; Buffer A = 0.1% FA/H_2_O; Buffer B = 0.1% FA in 80% Acetonitrile). All solvents used were of liquid chromatography–mass spectrometry grade. Spectra were acquired using XCalibur, version 2.3 (Thermo Scientific). A “top speed” data-dependent MS/MS analysis was performed. Dynamic exclusion was enabled with a repeat count of 1, a repeat duration of 30 sec, and an exclusion duration of 60 sec. Tandem mass spectra were extracted from Xcalibur “RAW” files, and charge states were assigned using the ProteoWizard 2.1.x msConvert script using the default parameters. Following this, the fragment mass spectra were searched against the Rattus SwissProt_2018_11 database using Mascot (Matrix Science, London, United Kingdom; version 2.4). The default probability cut-off score was used here. The search variables that were utilized were 10 ppm mass tolerance for precursor ion masses and 0.5 Da for product ion masses; digestion with trypsin; a maximum of two missed tryptic cleavages; and variable modifications of oxidation of methionine and phosphorylation of serine, threonine, and tyrosine. Cross-correlation of Mascot search results with X! Tandem was accomplished with Scaffold (version Scaffold_4.8.7; Proteome Software, Portland, OR, USA). The Scaffold program was used to determine the probability assessment of peptide assignments and protein identifications. Only peptides with ≥ 95% probability were considered. Reported peptide false discovery rates (FDR) rates from Scaffold ranged from 0.1–0.2%.

#### 4.3.3. Label-Free Quantitative Proteomics 

Progenesis QI for proteomics software (version 2.4, Nonlinear Dynamics Ltd., Newcastle upon Tyne, United Kingdom) was used to execute ion intensity-based label-free quantification as previously described [50]. Of the detected peptides, for further analysis, we selected those peptides with an expression difference between the groups using a significance of *p* < 0.05, as determined by Progenesis.

The mass spectrometry proteomics data were deposited to the ProteomeXchange Consortium via the PRIDE partner repository (https://www.ebi.ac.uk/pride/login) with the dataset identifier PXD020551 and 10.6019/PXD020551. The reviewer account details are Username: reviewer18943@ebi.ac.uk and Password: yCk26mos

### 4.4. Bioinformatics Analysis

The exported normalized peptide abundance values were analyzed and visualized in Perseus, version 1.6.2.3 [51,52]. Z-scores were calculated from the data by determining the mean of each column and then deducting it from each value in the column. The result was then divided by the standard deviation of the row or column to give the final Z-score. Unsupervised hierarchical clustering for the rows was executed to generate a visual heat map (parameters: distance = Euclidian; linkage = average; maximal numbers of clusters = 300). Principal component analysis (PCA) was carried out using the Orange data mining toolkit. Database for Annotation, Visualization, and Integrated Discovery (DAVID) was used for Gene Ontology (GO) annotation of the proteins for cellular component (CC), biological processes (BP), and molecular function (MF). The pathways affected were visualized by KEGG (Kyoto Encyclopedia of Genes and Genomes).

### 4.5. Statistics

Statistical analysis for the quantitative mass spectroscopic data was performed by the Progenesis software using one-way ANOVA to determine if treatment groups were significantly different from the control group. Following this, analysis of the individual proteins was performed using the GraphPad Prism software (Version 8.3.1) by either the unpaired *t*-test or the Mann–Whitney test. *p* < 0.05 was considered to be statistically significant.

## 5. Conclusions

This study gives a proteomic insight into the control by mitochondrial complex III in mitochondrial energetics and metabolic reprogramming. With complex III inhibition, we found significant disturbances in proteins that control several major processes within the mitochondria, such as fatty acid oxidation, TCA cycle, ETC, amino acid metabolism, and mitochondrial protein detoxification (Figure 4). We therefore propose that a decline in these processes could potentially trigger a glycolytic switch to initiate metabolic reprogramming, leading to sustained vasoconstriction and uncontrolled proliferation, which are key factors in the development of PAH. In addition to this, on the basis of our previous observations both in the animal model and in cell culture, we found a significant activation of the glycolytic pathway with Antimycin A treatment [6]. Therefore, targeting the proteins in the glycolytic pathway or increasing mitochondrial biogenesis and quality would also be a potential strategy to alleviate mitochondrial dysfunction-induced metabolic switch. Our observations suggest specific potential targets in the fatty acid and amino acid metabolism pathways to control or reverse the glycolytic switch and metabolic dysfunction in mitochondria. These potential targets require further validation and need to be fully investigated for future studies. Additionally, this method was developed for a rapid analysis of the mitochondrial proteome and did not consider trace proteins that were possibly masked by probable lysosome occurrence in the samples.
